# Acquired rifamycin resistance among patients with tuberculosis and HIV in New York City, 2001–2023

**DOI:** 10.1016/j.jctube.2024.100429

**Published:** 2024-03-15

**Authors:** Felicia Dworkin, Alice V. Easton, Byron Alex, Diana Nilsen

**Affiliations:** New York City Department of Health and Mental Hygiene, 42-09 28th St., Long Island City, NY, 11101-4132, United States

**Keywords:** Antimycobacterial resistance, Tuberculosis, HIV, Tuberculosis treatment, Acquired resistance

## Abstract

**Introduction:**

Acquired rifamycin resistance (ARR) in tuberculosis (TB) has been associated with HIV infection and can necessitate complicated TB treatment regimens, particularly in people living with HIV (PLWH). This work examines clinical characteristics and treatment outcomes of PLWH who developed ARR from 2001 to 2023 in New York City (NYC) to inform best practices for treating these patients.

**Methods:**

PLWH who developed ARR 2001–2023 were identified from the NYC TB registry.

**Results:**

Sixteen PLWH developed ARR; 15 were diagnosed 2001–2009 and the 16th was diagnosed in 2017. Median CD4 count was 48/mm^3^. On initial presentation, 14 had positive sputum cultures; of these, 12 culture-converted prior to developing ARR. Ten patients completed a course of TB treatment but subsequently relapsed; in six of these cases, ARR was discovered upon relapse, triggering treatment with a non-rifamycin-containing regimen, while in the other four, ARR was discovered during a second round of rifamycin-containing treatment. Three patients were lost to follow-up during their initial course of TB treatment and later returned to care; after being restarted on a rifamycin-containing regimen, ARR was discovered. Finally, three patients culture-converted during their first course of treatment but subsequently had cultures that grew rifamycin-resistant *Mycobacterium tuberculosis* prior to treatment completion, leading to changes in their treatment regimens. Among the 16 patients, eight died before being cured of TB, seven successfully completed treatment, and one was lost to follow-up.

**Conclusions:**

PLWH should be monitored closely for the development of ARR during treatment for TB, and sputum culture conversion should be interpreted cautiously in this group. Collecting a final sputum sample may be especially important for PLWH, as treatment failure and relapse were common in this population. The decrease in the number of cases of ARR among PLWH during the study period may reflect the decrease in the total number of PLWH diagnosed with TB in NYC in recent years, improved immune status of PLWH due to increased uptake of antiretroviral drugs, and improvements in the way anti-TB regimens are designed for PLWH (such as recommending daily rather than intermittent rifamycin dosing).

## 1 Introduction

Globally in 2021, 6.7 % of the 10.6 million estimated new cases of tuberculosis (TB) occurred among people living with HIV (PLWH), with 187,000 deaths in this population [1]. Patients can be treated for both TB and HIV at the same time, but concerns about drug-drug interactions and the potential for acquired rifampin resistance (ARR) can complicate TB treatment. ARR occurs when a patient who initially has rifampin (RIF)-susceptible TB develops RIF-resistant TB, which often requires a complicated and prolonged course of treatment.

Drug-drug interactions between some classes of antiretroviral therapy (ART) and RIF mean that many PLWH need to be treated for TB with regimens that do not contain RIF [2]. Rifabutin (RBT) is often recommended instead of RIF in TB regimens for PLWH. As the knowledge base has grown, guidelines for the treatment of TB in PLWH (including when and how to use rifamycins, with a focus on moving away from intermittent dosing) have been updated [3], [4], [5], [6].

Several studies and case reports have documented PLWH developing ARR during or after TB treatment [7], [8], [9], [10], [11], [12]. Studies in New York City (NYC) have suggested that relapse with ARR among PLWH may be associated with intermittent rifampin dosing during the intensive phase of treatment or poor compliance with therapy [13], [14]. A study of patients diagnosed with TB in California between 1994 and 2006 found that HIV infection was associated with acquired drug resistance [15]. Analysis of data from four randomized controlled trials conducted in India between 1999 and 2013 found that, among patients treated with a thrice-weekly TB regimen, PLWH were at a higher risk of ARR, in the presence of baseline isoniazid resistance [16].

This study examines the clinical characteristics of patients living with HIV, who were diagnosed with TB between 2001 and 2023, who developed RIF resistance during or after treatment with a rifamycin-containing regimen. This further elaborates on the development of ARR initially described in NYC patients from 1997 to 2000 [13] and better reflects the era of evolving ART therapy [17]. It is important to understand what factors can lead to the development of ARR in PLWH in order to treat PLWH in a way that will minimize the likelihood that they will have a TB relapse with a RIF-resistant infection.

## 2 Methods

### 2.1 Data Collection/Analysis

This is a retrospective case series describing NYC patients with TB (diagnosed between 2001 and 2023) who were coinfected with HIV and developed ARR either during TB therapy or following a completed course of TB treatment. Patients were identified from a review of the NYC TB electronic disease surveillance and case management system (Maven 5.4., Conduent Inc., Florham Park, NJ). Patients were included if they were living with HIV, had culture positive TB initially susceptible to RIF or RBT with or without other drug resistance, and were treated in any combination of NYC facilities (hospital, private clinic, or Health Department Chest Center). We defined patients as having developed ARR if a subsequent isolate was identified as RIF- or RBT-resistant after receiving a rifamycin-based regimen for at least 15 days. Data from patients’ clinical charts and the surveillance and case management system were collected using an abstraction form to obtain demographic, treatment, duration of therapy, compliance, resistance patterns, and patient outcomes to subsequent TB episodes (Supplementary Materials). ART information was abstracted as either having been on ART or not having been on ART during the period the patient was treated for TB.

TB culture and drug susceptibility testing (DST) were performed according to standard lab protocols. Whether a second episode of TB disease was likely due to a new infection or a relapse of the prior infection was based on comparing spacer oligonucleotide type (spoligotype) and IS6110 restriction fragment length polymorphism (RFLP) from initial and subsequent specimens.

### 2.2 Definitions

An episode of TB was defined as the period from diagnosis of active TB disease through treatment completion, treatment failure, loss to follow-up, or death. The date of diagnosis of ARR was defined as the date of identification of a rifamycin-resistant isolate by phenotypic susceptibility testing. Culture conversion was defined as two negative cultures taken at least a month apart. Treatment failure was defined as a positive *M. tuberculosis* culture result after the patient received appropriate TB treatment for at least four months. Treatment completion for rifamycin-resistant TB was defined as at least 18 months of an appropriate regimen with at least 12 months of treatment following the last negative culture, where appropriate treatment was defined as treatment with at least two drugs to which the isolate was known to be susceptible. Relapse was defined as the growth of *Mycobacterium tuberculosis* from any anatomical site after the completion of treatment.

### 2.3 Ethics

The New York City Department of Health and Mental Hygiene (NYC DOHMH) Institutional Review Board determined that this project (Protocol #11–021) was approved pursuant to 45 CFR §46.110(b)(1)(category F5). This study involves materials that were collected for non-research purposes, and the IRB determined that the study poses minimal risk to participants.

This project involves only data routinely collected as part of normal program services. Consent was not obtained for this secondary research analysis as the research activities only involved information that is regulated for public health purposes. This work was supported by New York City Department of Health and Mental Hygiene Bureau of Tuberculosis Control program funds. The authors had access to all the data in the study and had final responsibility for the decision to submit for publication. The authors declare no competing interests.

## 3 Results

From 2001 to 2023, 16,637 individuals were diagnosed with TB in NYC. Of these, 1683 (10 %) were among PLWH, though 3720 (22 %) of individuals did not have a documented HIV status. Twenty patients developed ARR during this time period; four were excluded as HIV status was either negative or unknown. The remaing 16 cases were among PLWH. All 16 patients had low CD4 counts (median 48/mm^3^); 10 were on ART at some point during TB treatment (Table S1).

Fifteen of the ARR cases among PLWH were identified during 2001–2009. During this period, there were 8310 total TB cases in NYC, of which 1186 (14 %) were among PLWH. Of those 1186, 921 (78 %) completed TB treatment, and 200 (17 %) died before or during treatment. Only one case of ARR among PLWH was identified during 2010–2023, a period when there were 8327 new cases of TB in NYC, of which 497 (6 %) were among PLWH. Of these, 386 (78 %) completed TB therapy by 02/09/2024, and 49 (10 %) died before or during treatment.

At initial diagnosis, five of the 16 PLWH with ARR had pulmonary TB, two had extrapulmonary TB, and nine had both pulmonary and extrapulmonary TB. Among the 14 patients with pulmonary involvement, initial chest x-rays showed cavitary disease for one patient, non-cavitary abnormal findings for 12 and normal findings for one (Table S1).

Twelve patients were male and four were female, with a median age of 43 years (range 19–53 years) at initial diagnosis of ARR by phenotypic DST. Eleven were Black and non-Hispanic, three were White and Hispanic, and two were Asian and non-Hispanic. Nine patients reported a history of substance abuse, with eight using drugs within 12 months of TB diagnosis.

During their initial TB episodes, 11 of the patients had TB that was susceptible to all first-line anti-TB drugs, four had isoniazid resistance (A, E, F, K), and one had streptomycin resistance (I). Genotyping data were available for all 16 initial TB episodes. Thirteen patients had genotyping data available for subsequent episodes (spoligotype for 13 and RFLP for 11), and in all 13 of these cases, the isolates from the initial and subsequent episodes matched.

All 16 patients were treated with a rifamycin-containing regimen. Two (G, J) did not tolerate their initial regimens and were changed to alternative medications. All patients had documented sputum culture conversion, except for two who had extrapulmonary TB only (I, M) and two who were lost prior to treatment completion (G, O). The median duration of TB treatment prior to diagnosis of ARR was 10 months (range < 1–30 months).

Ten patients completed a course of therapy but subsequently relapsed (Table 1, Fig. S1). Three patients were lost to follow-up during their initial course of treatment for TB and later returned to care (G, M, O). After being restarted on a rifamycin-containing regimen, ARR was discovered. Finally, three patients culture-converted during their first course of treatment but subsequently had cultures that grew rifamycin-resistant *M. tuberculosis* prior to treatment completion, leading to changes in their treatment regimens (A, J, L).

**Table 1:** Treatment of Initial Episode of TB (n = 16).

Patient	Initial Drug Resistance*	TB Medications* (†)	Rifamycin Dosing (†)	Frequency of Directly Observed Therapy (†)	Overall Compliance with Directly Observed Therapy (†)	Months on Treatment	Sputum Culture Conversion	Outcome of Treatment
**A**	H-resistant	H, Rifamycin, Z, E	RBT 150 mg QD (4)in continuation phase	None	Hospital supervised	12	Yes	Treatment failure
**B**	Susceptible	H, Rifamycin, Z, E	RBT 300 mg QD (5);	Intermittent: RBT 150 mg TIW (7)	Hospitalsupervised	13	Yes	Completed;Relapsed after 6 months
			RBT 150 mg TIW (7)					
**C**	Susceptible	H, Rifamycin, Z, E	RBT 300 mg QD	None	Hospital supervised	14	Yes	Completed;Relapsed after 3 months
**D**	Susceptible	H, Rifamycin, Z, E	R 600 mg QD (4);	Intermittent: RBT 150 mg TIW (3)	Hospital supervised	7	Yes	Completed;Relapsed after 5 months
			RBT 150 mg TIW (3) in continuation phase					
**E**	H-resistant	H, Rifamycin, Z, E	RBT 300 mg QD (7);	Intermittent: RBT 300 mg BIW (3)	77 % (3)	10	Yes	Completed;Relapsed after 6 months
			RBT 300 BIW (3)					
**F**	H-resistant	Rifamycin, Z, E	R 600 mg QD	Daily	60 % (10)	10	Yes	Completed; Relapsed after 5 months
**G**	Susceptible	H, R, Z, E (5);	R 600 mg QD	Daily	66 % (11)	11	No	Lost after 11 months (Treatment failure)
		R, LEV (2);R, E, LEV (4)						
**H**	Susceptible	H, Rifamycin, Z, E	R 600 mg TIW (5)	Intermittent: R 600 mg TIW (5)	77 % (5)	5	Yes (Sputum culture performed only once)	Completed;Relapsed after 3 months
**I**	SMN-resistant	H, Rifamycin, Z, E	R 600 mg QD	Daily	83 % (7)	7	Extrapulmonary Only	Completed;Relapsed after 3 months
**J**	Susceptible	H, RBT, Z, E (1);	RBT 300 mg QD (1);	Intermittent: RBT 150 mg TIW (13)	99 % (30)	30	Yes	Treatment failure
		E, SMN, MOX (3);	RBT 450 mg QD (2);					
		E, SMN (2); E, SMN, LEV (2);	RBT 150 mg QD (3);					
		RBT, E, Z, MOX (3);	RBT 150 mg TIW (13)					
		H, E, SMN, MOX, (4);	(Gaps with RBT)					
		H, RBT, E (13)						
**K**	H-resistant	Rifamycin, Z, E	R 600 mg QD (5);	Daily	93 % (10)	10	Yes	Completed;Relapsed after 4 months
			RBT 150 mg QD (5)in continuation phase					
**L**	Susceptible	H, Rifamycin, Z, E	RBT 150 mg QD (7);	Intermittent: RBT 150 mg TIW (1)	89 % (3)	11	Yes	Treatment failure
			RBT 150 mg TIW (1);					
			RBT 450 mg QD (5)					
**M**	Susceptible	H, Rifamycin, Z, E	R 600 mg QD	Daily	Self-administered	5	Extrapulmonary Only	Lost after 5 months (Treatment failure)
**N**	Susceptible	H, Rifamycin, Z, E	R 600 mg QD (1);	Intermittent: R 600 mg BIW/TIW (5)	100 % (7)	7	Yes	Completed;Relapsed after 4 months
			R 600 mg BIW (2);					
			R 600 mg TIW (3)					
**O**	Susceptible	H, R, Z, E	R 600 mg QD	None	Hospital supervised	<1	No	Lost after < 1 month;;(Treatment failure)
**P**	Susceptible	H, Rifamycin, Z, E	RIF 600 mg QD (0.5);	None/hospitalized (2)	97 % (5)	7	Yes	Completed;Relapsed after 15 months
			RBT 300 mg QD (6.5);	Daily (2)				
				5x/week (3)				

* H: isoniazid; R: rifampin; Z: pyrazinamide; E: ethambutol; RBT: rifabutin; LEV: levofloxacin; SMN: streptomycin; MOX: moxifloxacin

† Duration in months; QD = every day, BIW = twice a week, TIW = three times a week.

### 3.1 Patients who completed treatment for their initial episode of TB (n = 10)

Five patients who relapsed (C, F, I, K, P) were on a daily rifamycin-based regimen for 7–14 months; four of these were on directly-observed therapy (DOT) (three with at least 80 % of the total expected doses taken under direct observation) and one was on hospital-supervised treatment. The remaining five patients who relapsed (B, D, E, H, N) were on an intermittent rifamycin-containing regimen for 3–7 months. Three of these patients were on DOT (one with 77 % and one with 100 % of doses observed), and two were on hospital-supervised treatment. Among these ten individuals, five (B, C, D, K and P) were on ART at some point during their TB treatment. For nine of these patients, relapse occurred after treatment completion for their first episode of TB. For one patient (N), ARR was found after he completed treatment for a second episode of drug-susceptible TB.

### 3.2 Treatment failure during first episode of TB (n = 6)

Six patients experienced a failed course of treatment for their initial TB episode. Three of these patients (G, M, O) were lost during treatment; one had 66 % compliance with DOT and culture conversion was never documented; one was on a regimen that included self-administered rifampin daily for extrapulmonary disease; and the third had hospital-supervised daily therapy for less than a month, then continued self-adminstered treatment with a regimen that included daily rifampin. The other three patients (A, J, L) had sputum culture conversion, but later developed positive cultures in sputum (A, L) or CSF (J) that were resistant to rifampin while still on therapy for their initial TB episode (at 12, 30, and 11 months, respectively). Of note, patient J had pulmonary and meningeal TB and was treated continually with multiple different regimens due to drug intolerance for 30 months before developing ARR. This patient initially converted both sputum and CSF cultures, but developed new positive pansusceptible *M. tuberculosis* CSF cultures six months into therapy. During the next 24 months of treatment, the patient’s CSF culture-converted, and the patient received an additional 16 months of therapy with rifabutin three times a week with high DOT compliance before developing ARR in the CSF only. Five of these six individuals were on ART at some point during their TB treatment Table 2, Table 3.

**Table 2:** Treatment of Second Episode of TB (n = 16).

Patient	Drug Resistance*	TB Medications* (†)	Rifamycin Dosing (†)	Overall Compliance with Directly Observed Therapy (†)	Months on Treatment	Sputum Culture Conversion	Outcome of Treatment
**A**	H, R, RBT	RBT, E, Z, SMN, CYC, CIP	RBT (2)	Hospital supervised	27	Yes	Completed 24 months after culture conversion
**B**	R, RBT	H, E, Z, LEV, SMN		No therapy (4) while lost; Hospital supervised (17)	29	Yes	Completed 27 monthsafter culture conversion
**C**	R, RBT	H, Z, E, MOX, SMN, ETH		Hospital supervised	20	Yes	Completed 18 months after culture conversion
**D**	R, RBT	H, Z, E, MOX, AMN (3)		Hospital supervised	7	Yes	Died; Non-TB related (6 months after culture conversion)
**E**	H, R, RBT	Z, E, ETH, LEV, AMN (4)		93 % (7)	20	Yes	Died; Non-TB related (19 months after culture conversion)
**F**	H, R, RBT	Z, E, SMN, GAT, SMN, CYC		Hospital supervised	4	Yes	Died; Non-TB related (2 months after culture conversion)
**G**	R, RBT	H, R, Z (8);H, Z, E, CAP, MOX (25)	R 600 mg QD (8)	93 % (33)	33	Yes	Completed;Relapsed after 9 months
**H**	R, RBT	H, R, Z, E	R 600 mg QD (4)	Initially hospital supervised (2)	4	No	Died due to TB/HIV
**I**	R, RBT, SMN	H, E, Z, LEV		89 % (18) and hospital supervised	22	Yes	Died; Non-TB related (22 months after culture conversion)
**J**	R, RBT	H, RBT, Z, E, MOX (4);	RBT 150 mg TIW	Hospital supervised	19	CSF converted	Completed 18 months after culture conversion
		E, MOX, S (6);H, E, ETH, MOX (9)	(4) then discontinued				
**K**	H, R	E, MOX, Z, RBT (4);Z, E, RBT MOX, ETH, LNZ (12)	RBT 150 mg TIW	85 % (16)	24	CSF converted	Completed 20 months after culture conversion
			(10) then discontinued				
**L**	R, RBT	Z, E, RBT, ETH, LEV		Hospital supervised	1	No additional specimens	Died due to TB/HIV
**M**	R, RBT, K	H, R, Z, E	R 600 mg QD (1)	Hospital supervised	1	No additional specimens	Died due to TB/HIV
**N**	Susceptible	H, R, Z, E	R 600 mg QD (5); RBT 150 mg QD (2)	100 % (7)	7	Yes	Completed;Relapsed after 6 months
**O**	R, RBT	H, R, Z, E (1)	R 600 mg QD (1)	Hospital supervised	4	Yes	Lost after 4 months
		H, MOX, Z, E, CAP (3)					
**P**	R, RBT	H, AMN, PAS, RBT, Z, MOX, LNZ, E, CYC	RBT 300 QD (1)	None/hospitalized (1)	13	Yes	Completed 9 months after culture conversion
				5x/week (12)			
		H, PAS, RBT, Z, MOX, LNZ, E, CYC, CAP					
				98 % (approximate)			
		H, PAS, Z, MOX, LNZ, E, CYC, CAP					
		H, Z, MOX, LNZ, E, CAP					
		H, AMN, Z, MOX, LNZ, E					
		H, AMN, Z, MOX, E					
		H, AMN, Z, E, LEV					
		H, Z, E, LEV					

* H: isoniazid; R: rifampin; Z: pyrazinamide; E: ethambutol; RBT: rifabutin; LEV: levofloxacin; SMN: streptomycin; MOX: moxifloxacin; CYC: cyclosporine; CIP: ciprofloxacin; AMN: amikacin; ETH: ethionamide; GAT: gatifloxacin; CAP: capreomycin; LNZ: linezolid; K: kanamycin

† Duration in months; QD = every day, BIW = twice a week, TIW = three times a week.

**Table 3:** Treatment of Third Episode of TB.

Patient	Drug Resistance*	TB Medications* (†)	Rifamycin Dosing (†)	Overall Compliance with Directly Observed Therapy (†)	Months on Treatment	Sputum Culture Conversion	Outcome of Treatment
**G**	Susceptible	H, Z, CAP (2);	R 600 mg QD (2)	Hospital supervised	15	Yes	Died; Non-TB related causes (14 months after culture conversion)
		H, R, Z, LEV (2)	RBT 300 mg QD (7)				
		H, E, MOX (3); H, RBT, Z, E (7)					
**N**	R, RBT	H, E, Z, SMN, MOX		98 % (27)	27	Yes	Completed 25 months after culture conversion

*H: isoniazid; R: rifampin; Z: pyrazinamide; E: ethambutol; RBT: rifabutin; LEV: levofloxacin; SMN: streptomycin; MOX: moxifloxacin; CAP: capreomycin

† Duration in months; QD = every day, BIW = twice a week, TIW = three times a week.

### 3.3 Treatment outcomes of subsequent episodes of TB

Fifteen patients developed ARR during their 2nd episode of TB and the 16th patient (N) developed ARR during a 3rd episode of pulmonary and hematological *M. tuberculosis* disease. Patient G had a 3rd episode of disease in which the isolate tested was susceptible to all drugs, despite tests having detected ARR in the prior episode. This could have resulted from multiple *M. tuberculosis* populations simultaneously infecting that individual, or from a test error; the potential for resistance to RIF during the 3rd episode was kept in mind when this patient’s treatment regimen was being designed. In both cases with three episodes, isolates from all three episodes matched by genotype.

Four patients (A, E, F, K) initially treated for isoniazid-resistant disease developed multidrug-resistant (MDR) TB after acquiring rifamycin resistance. Two of these (E, F) died of non-TB related causes after sputum cultures converted to negative. The other two patients (A, K) completed 24 and 20 months of treatment after sputum and CSF culture conversion, respectively.

Registry data showed that, of the eight patients who died, three (H, L, M) were considered to have had TB-related deaths, due to a lack of sputum culture conversion. The other deaths were considered non-TB related because they occurred while patients were in the continuation phase of ARR therapy and had culture-converted.

The seven remaining patients, of whom six completed TB therapy and one (O) was lost, were not reported again to the BTBC registry through 2023, nor was a record of death filed with the Bureau of Vital Statistics through 2023.

## 4 Discussion

During 2001–2023, ARR TB occurred in 0.1 % (20/16637) of all patients with TB in NYC, and in 1 % (16/1683) of patients with TB and HIV coinfection. Similarly, a previous study in NYC (1997–2000) reported that 0.9 % of patitents with TB and HIV developed ARR [13]. In the current study of 16 cases of ARR in PLWH, all patients had low CD4 counts at some point during TB treatment. Nine were on daily rifamycin therapy throughout the first episode, six received daily rifamycins only during the intensive phase and intermittent rifamycins during the continuation phase, and one was on intermittent rifamycin therapy throughout treatment. Four of the 16 initially had isoniazid-resistant TB and developed MDR TB after acquiring rifamycin resistance; two of these patients died and two completed treatment. Genotyping confirmed the same strain in all but three patients (for whom genotyping results were not available) for subsequent episodes of TB disease.

Sputum culture conversion was documented for the majority of patients (12 of 14) who presented with positive sputum cultures during their initial TB episode. Although culture conversion is usually used as a proxy for end-of-treatment outcomes, others have found that sputum culture conversion in HIV-infected MDR TB patients is not a robust marker [18]. Thus, PLWH should be monitored closely for the development of ARR during treatment for TB, and sputum culture conversion should be interpreted cautiously in this group. Collecting a final sputum sample may be especially important for PLWH, as treatment failure and relapse were common in this population

Although our study did not directly assess risk factors for development of ARR, our findings are consistent with other reports of ARR being associated with HIV infection, initial resistance to isoniazid, low CD4 counts, and intermittent TB therapy during the continuation phase [3], [15], [19], [20]. Notably, a high proportion of patients in the current study received daily rifamycins during the intensive phase, followed by either daily or intermittent rifamycins. Changes in TB treatment recommendations for all patients, and for PLWH in particular, likely contributed to the decline in cases of ARR seen in NYC since 2009. Intermittent rifamycin dosing is now avoided, with treatment plans specifying a daily dosage of rifamycin. Our findings also show that, among patients with ARR who completed an adequate course of therapy, including those with MDR TB, there have been no further relapses or deaths identified.

### 4.1 Limitations

There are several limitations to this study. Only patients who were under care by providers in NYC were included. Those who developed ARR before presenting or after leaving NYC are not included in these findings. Furthermore, the results of any clinical follow-up that occurred outside of NYC was usually not shared with the NYC DOH. While all patients had low CD4 counts (median 48/mm^3^) and several were on ART at some time during TB therapy, it was not possible to determine when the ART was taken during TB treatment for most individuals based on retrospective chart review. It is possible that the CD4 count improved during TB therapy, but this information was not available. Additionally, therapeutic drug level monitoring was not performed as it was not the standard of practice for patients with susceptible or isoniazid-resistant TB, unless otherwise indicated, as per recommendations [21], [22]. It is possible that the use of intermittent therapy or drug-drug interactions with ART caused a lower serum drug level of rifamycins, contributing to the development of ARR [23], [24].

A final limitation is that the methods used to identify rifamycin resistance have changed during the study period; newer tests provide greater sensitivity and quicker detection of resistance [25], [26], [27]. It is possible that some of the earlier patients in this study, whose infections were originally characterized as rifamycin-sensitive, had low-level rifamycin resistance that went undetected at first. Thus, some of the decrease in ARR could be due to modern diagnostics identifying low-level rifamycin resistance at the start of treatment.

Increased availability of molecular methods may mean that low-level rifamycin resistance is detected sooner, possibly leading some patients to have their infection categorized at rifamycin-resistant from the start, and thus not as ARR at a later date. This development is a benefit overall: it means that resistance can be identified more quickly and with greater sensitivity, which could in turn lead to better treatment outcomes and less transmission.

As the burden of TB continues to decline and treatment and prevention modalities for HIV improve and evolve, the incidence of ARR in NYC has continued to decrease. In particular, the decrease in the number of cases of ARR among PLWH during the study period (with only one case since 2010) may reflect the decrease in the total number of PLWH diagnosed with TB in NYC in recent years, and improved immune status of PLWH due to increased use and effectiveness of antiretroviral drugs [28]. Since 2001, most patients with TB and HIV in NYC have experienced cure of their TB. Though ARR has become rare among PLWH in recent years, it is important to monitor for its development among PLWH, even after culture conversion. In NYC, working with HIV care providers to design TB treatment regimens that minimize the likelihood of ARR (such as regimens with daily rather than intermittent rifamycind dosing) and supporting patients during treatment have been important steps toward maintining the downward trend in ARR in NYC.

Author contributions

Felicia Dworkin: conceptualization, methodology, formal analysis, resources, data curation, writing – original draft, project administration. Alice V. Easton: formal analysis, validation, data curation, writing – review and editing. Byron Alex: conceptualization, methodology, formal analysis, resources, data curation, writing – original draft. Diana Nilsen: conceptualization, resources, data curation, writing– review and editing, supervision.

## CRediT authorship contribution statement

**Felicia Dworkin:** Writing – original draft, Resources, Project administration, Methodology, Formal analysis, Data curation, Conceptualization. **Alice V. Easton:** Writing – review & editing, Validation, Formal analysis, Data curation. **Byron Alex:** Writing – original draft, Resources, Methodology, Formal analysis, Data curation, Conceptualization. **Diana Nilsen:** Writing – review & editing, Supervision, Resources, Data curation, Conceptualization.

## Declaration of competing interest

The authors declare that they have no known competing financial interests or personal relationships that could have appeared to influence the work reported in this paper.
