# Supplementary material for: Acquired rifamycin resistance among patients with tuberculosis and HIV in New York City, 2001–2023
Source: J Clin Tuberc Other Mycobact Dis. 2024 Mar 15;35:100429. doi: 10.1016/j.jctube.2024.100429 (PMC10979258; doi:10.1016/j.jctube.2024.100429)

**Supplementary materials:**

Chart Extraction Form for Patients with Relapse (RIF-resistant)

Name_________________________________ CRS #__________________

Date of Birth____________________________

Gender___________________________________

COB: ___________ Year Entered US:__________

Illicit Drug use?

Alcohol use?

Hepatitis?

Diabetes?

Patient’s weight_________________________

Other medical conditions____________________________

Known Renal Insuffiency (y/n)_______________________

List of all Medications________________________________

HIV status (y/n)__________

HIV medications:___________________________________________________

Dosages_________________________________________________________

Date of therapy started:______________________________________________

Non- HIV meds:___________________________________________________

Date of therapy started:______________________________________________

(For each treatment episode fill out the following)

**Treatment Episode #** _____

Diagnosis site: Hospital, DOHMH clinic, Private Provider

Therapy given by: Hospital, DOHMH clinic, Private Provider

Commisioners’s orders? D1, D2, D3,D4, D5

Date of diagnosis: __________________

Is this initial site of TB Disease? y/n

Initial Site**(s)** of TB Disease_______________________________________

Secondary sites? ________________________________

If pulmonary, cavitary (y/n)?__________________________________

Culture negative after 2 months (y/n)?_______________________________

Drug Resistance other than RIF (y/n)?________________________________________

If yes, resistant to:______________________________

Weeks on therapy till culture conversion:______________________

Dates of therapy:_________________________________

Total Weeks Treatment for TB Disease episode_________________________

TB Drug regimen:____________________________

How many weeks on this regimen?___________________

Change in Drug regimen:

How many weeks on this regimen?___________________

Use of Rifabutin during the initial phase? y/n or rifampin

Started when?__________________

Frequency of RBT given?_______

Use of Rifabutin during the continuation pahse? y/n or rifampin

Started when?__________________

Frequency of RBT given?_______

DOT (y/n)?_________________________________

Weeks on DOT?_______________________

Adherence less than 80% (y/n)___________________________

If yes, adherence % ____________________

Intermittent Therapy Use?_____________________________________

Intermittent Therapy 2 or 3 times per week?_________________________

Number of weeks on intermittent therapy?________________________

If intermittent therapy, companion drugs given intermittent (y/n)?___________________

Drugs in regimen:______________________________

Dosages?

Intermittent Therapy in the Intensive Phase?_____________________

Interruptions in therapy for more than one week? (y/n)______________________

If yes, weeks of interruption?________________

For HIV positive, CD4 count (at start of therapy)___________________________

Viral Load_____________________________________________________

CD4 at end of therapy___________ Viral Load at end of therapy_____________

**For each Relapsed episode fill out the above and additional items:**

Date of relapse?____________________

Number of weeks between last positive culture and relapse________________________

Number of weeks between completed therapy and relapse with new positive culture___________

Additional Resistance at Relapse (list resistant drugs)? ___________________________________________________________________

Therapeutic Drug Monitoring? (If yes, results)________________________________

Retreatment Regimen_____________________________________________________

Dates of retreatment regimen:_________________________________

Total Weeks Treatment for TB Disease episode_________________________

RBT use in retreatment regimen? y/n or Rifampin

Initial phase_______

Frequency given_______

Continuation phase________

Frequency given_______

Total months on RBT or RIF______

Total length of therapy_____

Patient died? y/n

Cause of death?

**Table S1: Patient Characteristics (n=16)**

| **Patient**  **Age*, Sex** | **Race/Ethnicity** | **Disease site**  **(per episode)** | **Initial  Chest  X-Ray** | **History of**  **Drug Abuse** | **CD4  (per mm^3^)** | **RFLP and  Spoligotype** | **ART**  **Received†** |
| --- | --- | --- | --- | --- | --- | --- | --- |
| **Patient A**  **50, Female** | Black/ Non-Hispanic | 1st: Pulmonary  2nd: Pulmonary | Non-cavitary | Yes‡ | 21 | C(3) S00030 § | Yes |
| **Patient B**  **46, Male** | Black/Non-Hispanic | 1st: Pulmonary, Blood, Genitourinary  2nd: Pulmonary | Non-cavitary | Yes‡ | 50 | CS104(11) S00074 § | Yes |
| **Patient C**  **40, Female** | White/Hispanic | 1st: Pulmonary, Lymphatic  2nd: Pulmonary, Blood | Normal | No | 46 | N4(15) S00034 § | Yes |
| **Patient D**  **43, Male** | White/Hispanic | 1st: Pulmonary, Lymphatic  2nd: Pulmonary, Genitourinary, Bone-joint | Non-cavitary | Yes‡ | 94 | CS31(12) S00074 | Yes |
| **Patient E**  **40, Male** | Black/ Non-Hispanic | 1st: Pulmonary  2nd: Pulmonary | Non-cavitary | Yes‡ | 49 | H(2) S00009 § | No |
| **Patient F**  **45, Male** | Black/ Non-Hispanic | 1st: Pulmonary, Pleural  2nd: Pulmonary | Non-cavitary | Yes‡ | 35 | BJ(12) S00050 § | No |
| **Patient G**  **19, Female** | Asian/ Non-Hispanic | 1st: Pulmonary  2nd: Pulmonary  3rd: Pulmonary, Meningeal | Cavitary | No | 228 | 001(19) S00931 § | Yes |
| **Patient H**  **48, Male** | Black/ Non-Hispanic | 1st: Pulmonary  2nd: Pulmonary | Non-cavitary | Yes‡ | 35 | NW(8) S00332 | No |
| **Patient I**  **53, Male** | Black/ Non-Hispanic | 1st: Lymphatic  2nd: Pulmonary, Lymphatic | Normal | Yes‡ | 172 | H(2) S00244 § | No |
| **Patient J**  **32, Male** | Asian/ Non-Hispanic | 1st: Pulmonary, Meningeal  2nd: Meningeal | Non-cavitary | Yes | 35 | IP25(12) S00480 § | Yes |
| **Patient K**  **43, Male** | Black/ Non-Hispanic | 1st: Pulmonary, Meningeal  2nd: Meningeal | Non-cavitary | Yes‡ | 49 | BW388(9) S00241 § | Yes |
| **Patient L**  **44, Male** | Black/ Non-Hispanic | 1st: Pulmonary, Blood, Bone-joint  2nd: Pulmonary | Non-cavitary | No | 2 | AH5(5) S01776 | Yes |
| **Patient M**  **50, Male** | Black/ Non-Hispanic | 1st: Gastrointestinal  2nd: Pulmonary | Non-cavitary | No | 84 | C(3) S00030 § | No |
| **Patient N**  **41, Male** | Black/ Non-Hispanic | 1st: Pulmonary  2nd: Pulmonary, Blood  3rd: Pulmonary, Blood | Non-cavitary | No | 43 | Y34(10) S00096 § | No |
| **Patient O**  **38, Female** | White/  Hispanic | 1st: Pulmonary, Blood, Gastrointestinal  2nd: Pulmonary, Disseminated | Non-cavitary | No | 3 | CC (13)  S00157 §¶ | Yes |
| **Patient P**  **40, Male** | Black/ Non-Hispanic | 1st: Pulmonary, Lymphatic  2nd: Pulmonary, Bone-joint | Non-cavitary | No | 20/ 274 | E11(8)  S01618 § | Yes |

* Age at initial diagnosis of acquired rifamycin resistance

† Abstracted from chart review as having been on antiretroviral therapy (ART) at any time during the period the patient was treated for TB

‡ Drug abuse within 12 months of initial TB diagnosis

§ Initial and resistant isolates matched by genotype

¶ One octal code spacer was deleted between the initial and resistant isolate; this was interpreted as a matching genotype

Figure S1. Patient outcomes by round of treatment.


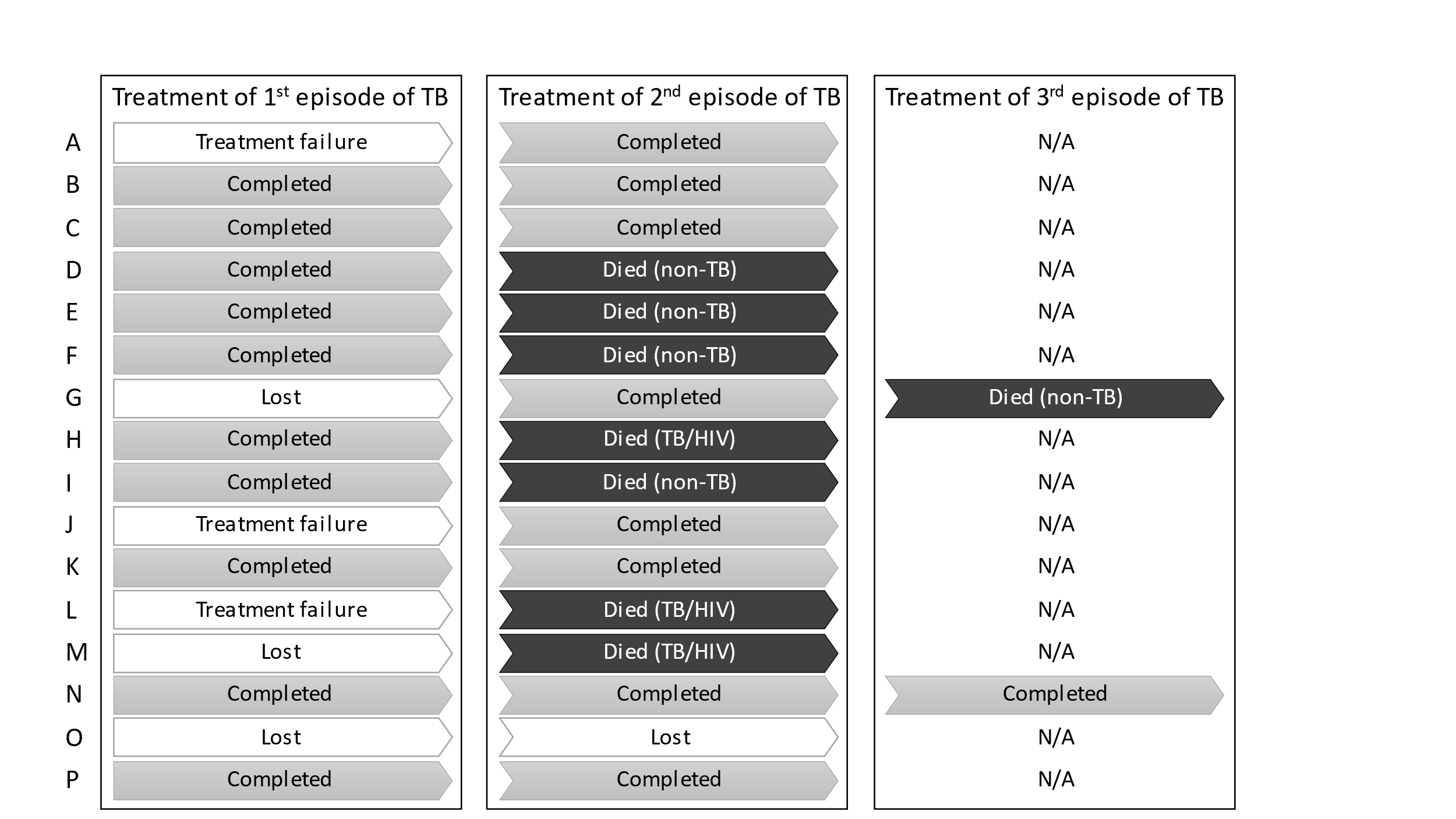

Supplement: Supplementary data 1 [file mmc1.docx]
